# Systemic Chemotherapy Is Modulated by Platelet-Activating Factor-Receptor Agonists

**DOI:** 10.1155/2015/820543

**Published:** 2015-04-02

**Authors:** Ravi P. Sahu, Matheus Ferracini, Jeffrey B. Travers

**Affiliations:** ^1^Department of Pathology and Laboratory Medicine, Indiana University School of Medicine, Indianapolis, IN 46202, USA; ^2^Department of Dermatology, Indiana University School of Medicine, Indianapolis, IN 46202, USA; ^3^Department of Pharmacology and Toxicology, Wright State University, Dayton, OH 45435, USA; ^4^Dayton Veterans Administration Medical Center, Dayton, OH 45435, USA

## Abstract

Chemotherapy is used to treat numerous cancers including melanoma. However, its effectiveness in clinical settings is often hampered by various mechanisms. Previous studies have demonstrated that prooxidative stressor-mediated generation of oxidized lipids with platelet-activating factor-receptor (PAF-R) agonistic activity induces systemic immunosuppression that augments the growth of experimental melanoma tumors. We have recently shown that treatment of murine B16F10 melanoma cells *in vitro* or tumors implanted into syngeneic mice and treated intratumorally with various chemotherapeutic agents generated PAF-R agonists in a process blocked by antioxidants. Notably, these intratumoral chemotherapy-generated PAF-R agonists augmented the growth of secondary (untreated) tumors in a PAF-R dependent manner. As both localized and systemic chemotherapies are used based on tumor localization/stage and metastases, the current studies were sought to determine effects of PAF-R agonists on systemic chemotherapy against experimental melanoma. Here, we show that systemic chemotherapy with etoposide (ETOP) attenuates the growth of melanoma tumors when given subsequent to the tumor cell implantation. Importantly, this ETOP-mediated suppression of melanoma tumor growth was blocked by exogenous administration of a PAF-R agonist, CPAF. These findings indicate that PAF-R agonists not only negatively affect the ability of localized chemotherapy but also compromise the efficacy of systemic chemotherapy against murine melanoma.

## 1. Introduction

Despite all available treatment options, the annual mortality rate of malignant melanoma is rapidly increasing in the United States [1, 2]. Although new immune based approaches such as anticytotoxic T lymphocyte antigen 4 (anti-CTLA-4) and antiprogrammed death 1 (anti-PD-1) have shown considerable promise in a subset of melanoma patients, systemic chemotherapy is still considered an option for advanced melanoma [3–5]. Several cellular resistance mechanisms exist that affect the clinical efficacy of chemotherapy against solid tumors including melanoma [6–9]. Of significance, chemotherapeutic agents due to their ability to induce the generation of reactive oxygen species (ROS) act as potent prooxidative stressors [10–12].

Several studies including ours have shown that prooxidative stressors, like ultraviolet B (UVB), generate the potent lipid mediator platelet-activating factor (PAF) and novel oxidized lipid glycerophosphocholines (Ox-GPCs) with PAF-R agonistic (or PAF-like) activity directly from cellular membrane phospholipids [13–17]. PAF and Ox-GPCs mediate their effects via binding to a seven-transmembrane-G-protein coupled receptor, the PAF-receptor (PAF-R), expressed on various immune and nonimmune cell types and cancer cells including melanoma [18–20]. PAF-R agonists/Ox-GPCs mediate systemic immunosuppression via cyclooxygenase type 2 (COX-2) dependent induction of the immunosuppressive cell type, regulatory T cells (Tregs), and the cytokine interleukin-10 (IL-10) [17, 21–24]. Importantly, our studies have shown that this PAF-R agonists-mediated systemic immunosuppression augments the growth of experimental cancers including melanoma and lung carcinoma in a PAF-R dependent fashion [25, 26].

Among various notable pathophysiological functions including cancer growth, angiogenesis, and metastases, PAF has been reported to modulate the effectiveness of chemotherapeutic agents [27, 28]. Importantly, our recent studies have provided compelling evidence that chemotherapeutic agents such as melphalan, etoposide (ETOP), dacarbazine, and cisplatin generate PAF-R agonists from PAF-R deficient murine B16F10 melanoma cells in a time and dose dependent manner and that this effect was pronounced in B16F10 cells expressing the PAF-R [29]. In a dual tumor model, where two melanoma tumors were implanted into syngeneic mice and only one tumor was treated intratumorally with chemotherapeutic agents (melphalan or ETOP) either in syngeneic mice expressing or deficient in the PAF-R, we demonstrated that chemotherapy-generated PAF-R agonists augmented the growth of secondary tumors in a PAF-R dependent manner [29]. Notably, this effect was blocked by systemic administration of antioxidants, COX-2 inhibitors, and neutralizing antibodies against Tregs, indicating an importance of the PAF/PAF-R signaling in potentially offsetting the therapeutic efficacy of intratumoral (localized) chemotherapy against murine melanoma [29]. In addition, perfusates collected from patients undergoing isolated limb chemoperfusion with melphalan chemotherapy for melanoma tumors residing in extremities exhibited high levels of PAF-R agonists, indicating that chemotherapeutic agents generate PAF-R agonists in human subjects [29].

Of significance, both localized and systemic chemotherapy are explored for melanoma patients based on the tumor localization, its stage, and metastases [4, 5, 30–32]. Given the findings of our study that PAF-R agonists can modulate an efficacy of intratumoral chemotherapy for experimental melanoma, its role in systemic chemotherapy needs to be elucidated. Our current studies demonstrate that systemic chemotherapy suppresses the growth of experimental melanoma tumors when started subsequent to the tumor cell implantation and that this effect is blocked by administration of the PAF-R agonist, carbamoyl-PAF (CPAF). These studies suggest that exogenous PAF-R agonists can subvert systemic chemotherapy.

## 2. Materials and Methods

### 2.1. Reagents and Cell Lines

All chemicals were obtained from Sigma-Aldrich unless indicated otherwise. Murine melanoma B16F10 and lymphoma EL4 cells were procured from ATCC and grown in DMEM high glucose media supplemented with 10% fetal calf serum and 100 *μ*g/mL mixture of penicillin and streptomycin.

### 2.2. Mice

Female C57BL/6-wild-type mice (PAF-R expressing; age 7-8 weeks) were purchased from the Charles River Laboratories. All mice were housed under specific pathogen-free conditions. All procedures were approved by the Institutional Animal Care and Use Committee of Indiana University School of Medicine.

### 2.3. *In Vivo* Tumor Growth Studies

To determine effects of systemic ETOP chemotherapy against experimental melanoma, 0.5 × 10^6^ murine B16F10 cells, which lack the functional PAF-R [25], were implanted subcutaneously (s.c.) into the shaved right hind flanks of syngeneic C57BL/6 (WT) mice. ETOP treatment at the dose of 36 mg/kg (dissolved in 100 *μ*L PBS with 0.5% DMSO) was started intraperitoneally (i.p.) either on day 6, day 3, or day 0 following tumor cell implantation and repeated every 3 days afterwards. Control mice received 0.5% DMSO in 100 *μ*L PBS i.p. as vehicle. The working dose of ETOP (36 mg/kg) was selected from our recently published studies [29]. To determine the effect of systemic PAF-R agonists on ETOP-mediated modulation of experimental melanoma tumor growth, WT mice were implanted with 0.5 × 10^6^ B16F10 cells s.c. followed by i.p. treatments with or without ETOP and PAF-R agonist, CPAF (nonmetabolizable carbamoyl-PAF) at a dose of 250 ng/mouse at days 0, 6, and 12 as per our published protocols [25]. Tumor growth (major and minor circumferences) was monitored and measured every 3 days before treatments with digital calipers (Mitutoyo Corp., Aurora, IL), and tumor volume was calculated (major circumference × minor circumference^2^/2). To assess whether systemic PAF-R agonists modulate tumor growth other than B16F10, 0.5 × 10^6^ murine EL4 lymphoma tumor cells were implanted s.c. into WT mice followed by i.p. injections of CPAF and measurement of tumor growth as described.

### 2.4. Statistical Analysis

All murine studies utilized at least four mice per experimental group and repeated as necessary to verify reproducibility and provide additional data for analysis. All statistical calculations were performed using GraphPad Prism 5. We used Student's* t*-tests for comparing two groups and one-way ANOVA with post hoc Bonferroni tests for more than two groups. The data represent mean values with SEM. Differences were considered statistically significant when the *P* value was less than 0.05.

## 3. Results and Discussion

Our recently published studies have demonstrated that chemotherapeutic agents generate PAF-R agonists from experimental murine B16F10 cells* in vitro* and* in vivo *[29]. Importantly, intratumoral (localized) chemotherapy-generated PAF-R agonists augmented the growth of preexisting secondary (untreated) melanoma tumors in a PAF-R dependent fashion [29]. Notably, these studies used a dual tumor model (two tumors were implanted but only one tumor was treated and the other tumor left untreated) to directly assess the ability of intratumoral chemotherapy mediated PAF-R agonists generation in the modulation of experimental melanoma tumor growth. As cancer patients can be subjected to numerous types of PAF-R agonists generating prooxidative stressors such as cigarette smoke and UVB radiation, the current studies were designed to test whether exogenous PAF-R agonists can modulate systemic chemotherapy. Although a combination of systemic chemotherapy and PAF-R antagonist has been shown to be effective in suppressing the growth of human melanoma tumors implanted in immune compromised nude mice [28], the role of PAF-R agonists in the modulation of systemic chemotherapy has not been studied.

**Figure 1:**
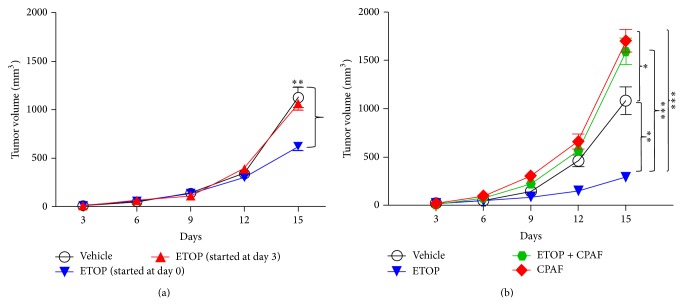
Modulation of B16F10 tumor growth by systemic chemotherapy with etoposide (ETOP) and the effect of PAF-R agonist (CPAF). (a) Murine B16F10 melanoma tumor cells (0.5 × 10^6^) were implanted into the shaved dorsal hind flanks of syngeneic C57BL/6-WT mice (5–7 mice/group) subcutaneously (day 0). Mice were treated with or without ETOP (36 mg/kg) intraperitoneally either at day 0, day 3, or day 6 repeated at every 3 days until the end of the experiment. Control mice received the vehicle (0.5% DMSO in 100 *μ*L PBS) by the same route. Tumor growth was monitored and measured with digital caliper and tumor volume (major circumference × minor circumference^2^/2) was expressed as mean ± SEM per group. Statistical differences (^∗∗^ *P* > 0.01) were noted between vehicle and ETOP treatment (day 0) at day 15. (b) Following subcutaneous B16F10 tumor cell implantation, C57BL/6-WT mice were treated with or without ETOP (36 mg/kg) intraperitoneally at day 0 repeated every 3 days until the end of the experiment. CPAF treatment (250 ng/mouse) was given intraperitoneally at days 0, 6, and 12. Control mice received the vehicle (0.5% DMSO in 100 *μ*L PBS) by the same route. Tumor growth was measured and tumor volume (major circumference × minor circumference^2^/2) was expressed as mean ± SEM per group and compared between the groups. Statistical differences were noted between (1) ^∗∗^ *P* < 0.01, vehicle and ETOP groups; (2) ^∗^ *P* < 0.05, vehicle and CPAF groups; and (3) ^∗∗∗^ *P* < 0.001, ETOP and CPAF and ETOP and ETOP + CPAF groups at day 15.

The first studies were designed to test if systemic chemotherapy with ETOP can attenuate experimental melanoma tumor growth in settings when ETOP treatments are given either subsequent to tumor cell implantation (day 0), after 3 days, or at day 6 (when more than half of the mice developed palpable tumors). To that end, PAF-R expressing C57BL/6-WT mice were implanted with PAF-R deficient murine B16F10 melanoma tumor cells followed by treatments with or without ETOP at day 0, day 3, or day 6 intraperitoneally repeated at every alternate 3 days until the end of the experiment. Our studies demonstrate that ETOP failed to suppress the growth of melanoma tumors when started at day 3 (Figure 1(a)) or day 6 (data not shown) as the differences in tumor volumes in ETOP versus vehicle-treated groups were not statistically significant. However, we observed a significant difference in the suppression of B16F10 tumor xenografts by ETOP treatment started at day 0 (Figure 1(b)). These studies are consistent with de Oliveira et al. studies demonstrating that dacarbazine only modestly suppressed the growth of B16F10 xenografts in C57BL/6 mice when started after 3 days of tumor cell implantation [28].

Our previous studies have shown that PAF-R agonists produced via various prooxidative stressors mediate systemic immunosuppression [17, 21–24, 33]. This systemic immunosuppression results in an augmentation of experimental melanoma tumor growth in a PAF-R dependent fashion in a process blocked by antioxidants, inhibitors of COX-2, or Tregs [25]. Notably, administration of PAF-R agonist, CPAF, mimicked prooxidative stressor-generated PAF-R agonist mediated effects [25]. Our next studies investigated if CPAF can modulate ETOP effectiveness in preclinical settings that resulted in significant suppression of experimental melanoma tumor growth (Figure 1(b)). To accomplish this, WT mice were implanted with B16F10 tumor cells followed by treatment with or without ETOP and CPAF as described by us previously [25]. We observed that CPAF-alone treated mice (used as a positive control) exhibited enhanced tumor growth compared to vehicle-treated mice (Figure 1(b)). Interestingly, a group of mice treated with both ETOP and CPAF resulted in augmentation of melanoma tumor growth similarly as seen in CPAF-alone treated group compared to ETOP and vehicle alone treated groups (Figure 1(b)). Notably, we did not observe any significant differences in murine weights with or without treatments in these studies (data not shown). These findings indicate that PAF-R agonists block ETOP effectiveness against murine melanoma. These studies are consistent with our recent studies assessing the role of intratumoral chemotherapy-generated PAF-R agonists in the modulation of B16F10 tumor growth using a dual tumor model [29]. We demonstrated that PAF-R agonists generated via intratumoral melphalan or ETOP chemotherapy by one tumor augmented the growth of second (untreated) tumor in a PAF-R dependent manner, indicating the importance of PAF/PAF-R signaling in attenuating the therapeutic efficacy of classical chemotherapeutic agents against murine melanoma [29]. These findings are in agreement with de Oliveira et al. studies demonstrating that systemic administration of PAF-R antagonist, WEB2170, significantly suppressed the growth of B16F10 tumors in C57BL/6 mice [28]. Similarly, these findings are consistent with studies by Seo et al., demonstrating that subcutaneous implantation of B16F10 cells mixed in Matrigel containing PAF augmented and ETOP suppressed the growth of experimental melanoma in C57BL/6-WT mice [34]. Notably, PAF blocked this ETOP-mediated attenuation of melanoma tumor growth in mice implanted with Matrigel mixed B16F10 cells containing PAF and ETOP [34]. These effects were mediated via NF-*κ*B-dependent upregulation of antiapoptotic Bcl-2 and Bcl-xL genes that resulted in an inhibition of ETOP-induced caspases 3, 8, and 9 activities [34]. Although these studies did not directly address the effects of systemic ETOP and PAF-R agonists in the modulation of experimental melanoma, they did support our findings that PAF-R agonists can attenuate ETOP chemotherapy effects against murine melanoma [34].

Notably, most melanomas express the PAF-R [27] and B16F10 cells do not [25]. Ectopic expression of the PAF-R in B16F10 cells resulted in similar increase in tumor xenografts by exogenous CPAF as in xenografts implanted with PAF-R deficient vector control cells in PAF-R expressing syngeneic mice [25]. This CPAF-mediated increase growth of PAF-R expressing and deficient tumor xenografts was not seen in PAF-R deficient mice, suggesting the role of host PAF-R versus tumoral PAF-R in mediating PAF-R agonists-induced increased tumor growth [25]. Nevertheless, PAF-R expression in B16F10 melanoma cells enhances chemotherapy mediated PAF-R agonists production compared to PAF-R deficient B16F10 melanoma cells [29]. In a similar line, studies by Onuchic et al. reported that in PAF-R expressing human SKmel37 melanoma cells, cisplatin treatment resulted in increased expression of the PAF-R and its accumulation [35]. Treatment with exogenous PAF protected SKmel37 cells from cisplatin-induced cell death. Moreover, systemic treatments with cisplatin or PAF-R antagonist, WEB2086, attenuated the growth of SKmel37 tumor xenografts substantially in nude mice and this effect was pronounced in a group of mice treated with a combination of cisplatin and WEB2086 [35].

**Figure 2:**
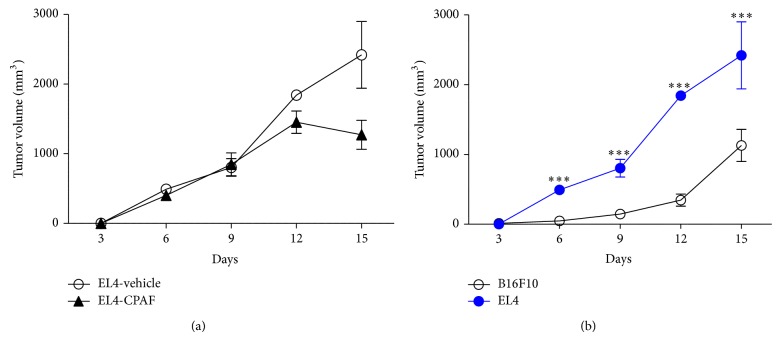
Effect of CPAF on EL4 tumor growth and comparison of basal B16F10 and EL4 tumor growth. (a) Murine syngeneic EL4 lymphoma tumor cells (0.5 × 10^6^) were implanted subcutaneously into the shaved dorsal hind flanks of C57BL/6 WT mice (4-5 mice/group). CPAF treatment (250 ng/mouse) was given intraperitoneally at days 0, 6, and 12. Tumor growth was measured and tumor volumes (major circumference × minor circumference^2^/2) were compared between the groups. (b) The basal growth rates between B16F10 and EL4 tumors were compared by assessing tumor volumes (major circumference × minor circumference^2^/2). Statistical differences were noted between EL4 and B16F10 tumors at day 6 (^∗∗∗^ *P* < 0.001), day 9 (^∗∗∗^ *P* < 0.001), day 12 (^∗∗∗^ *P* < 0.001), and day 15 (^∗∗^ *P* < 0.01).

Our previous studies have shown that systemic CPAF not only augments murine melanoma but also enhances murine epithelial Lewis Lung carcinoma (LLC1) tumor growth in C57BL/6 mice [26]. We next investigated if CPAF can affect the growth of murine EL4 lymphoma tumors. To that end, 0.5 × 10^6^ EL4 cells were injected s.c. into the flanks of C57BL/6 mice followed by treatment with or without CPAF as per our published reports [25, 26]. We demonstrate that CPAF did not modulate the growth of EL4 tumors as no significant difference in tumor volumes was noted between vehicle- and CPAF-treated mice (Figure 2(a)). The exact reason for this discrepancy is not clear at this time; however, we observed that the basal growth rate of EL4 tumors was two times faster than B16F10 tumors (Figure 2(b)) and thus CPAF has no additional tumor promoting effect. Of interest, this is likely not due to tumoral PAF-R expression as B16F10, LLC1, and EL4 tumor cells do not express PAF-R mRNA ([25, 26] and data not shown). This study does indicate that PAF-R agonists mediated effects are specific for certain tumor types.

## 4. Conclusions

Our current studies indicate that systemic ETOP chemotherapy against experimental melanoma is more effective when given simultaneous to the tumor cell implantation and chemotherapy-induced inhibition of tumor growth is blocked by exogenous PAF-R agonist. PAF-R agonist mediated effects are tumor specific as EL4 lymphoma tumors were not modulated by systemic CPAF as seen in B16F10 and LLC1 tumors. As many cancer patients also can be exposed to exogenous prooxidative stressors that are known to generate PAF-R agonists [17, 21–24, 33], these findings could have clinical implications in chemotherapy treatment failure.

## Abbreviations

ETOP: Etoposide
COX-2: Cyclooxygenase type 2
CPAF: 1-Hexadecyl-2-N-methylcarbamoyl glycerophosphocholine
PAF: Platelet-activating factor
PAF-R: PAF-receptor
ROS: Reactive oxygen species
Treg: Regulatory T cells.
